# Polydopamine-Laced Biomimetic Material Stimulation of Bone Marrow Derived Mesenchymal Stem Cells to Promote Osteogenic Effects

**DOI:** 10.1038/s41598-017-13326-y

**Published:** 2017-10-11

**Authors:** Dong Joon Lee, Yan-Ting Lee, Rui Zou, Renie Daniel, Ching-Chang Ko

**Affiliations:** 1 Oral and Craniofacial Health Sciences Research, School of Dentistry, University of North Carolina, CB #7455, Chapel Hill, NC 27599 USA; 2 Department of Oral and Maxillofacial Surgery, School of Dentistry, University of North Carolina, CB #7454, Chapel Hill, NC 27599 USA; 3 Department of Orthodontics, School of Dentistry, University of North Carolina, CB #7454, Chapel Hill, NC 27599 USA

## Abstract

A hydroxyapatite-collagen (HC) composite material can mimic composition and ultra-structures of natural bone and provide adequate bioactive material-tissue interactions. Incorporation of dopamine (DA) is one of keys in increasing the mechanical strength of the HC material to approaching that of cortical bone. In this study, the *in vitro* osteogenic effects of polydopamine-laced hydroxyapatite collagen calcium silicate (HCCS-PDA) were examined by culturing rat mesenchymal stem cells (rMSCs) on HCCS-PDA and HCCS coated plates. HCCS-PDA group demonstrated less cytotoxic from Live/Dead cytotoxic assay and displayed higher cell attachment, proliferation and mineralization than the HCCS group *in vitro*. For *in vivo* bone regeneration, HCCS-PDA or HCCS particulates with or without rMSC aggregates were implanted into rat critical-sized calvarial defects (CSD). After 12 weeks, calvarial bone regeneration was evaluated radiographically, histologically, and histomorphometrically. While the majority of new bone formation occurred around the HCCS-PDA particulates with rMSC aggregates, The HCCS-PDA particulates without rMSC aggregates showed limited osteoconductivity. HCCS with or without rMSC aggregates resulted in less bone formation, indicating a prominent role of DA in effective bone regeneration. Therefore, the HCCS-PDA biomaterial with the aid of rMSCs can be used to develop therapeutic strategies in bone tissue engineering with numerable clinical applications.

## Introduction

According to recent reports from the global bone graft substitute market, over 3 million musculoskeletal surgical procedures are performed in the United States annually^1^. The current clinical standard of care for damaged bone is surgical repair, though this poses drawbacks as every available surgical repair technique has its share of disadvantages. For example, autograft implantation is a technically demanding procedure associated with long surgical times, donor site morbidity accompanied by the need for a second surgical site^2^, and limited graft availability, especially in younger patients^3^. Nevertheless, the autograft from the iliac crest is the gold standard in current bone repair, primarily due to a lack of alternative graft material options. Studies have shown that poor bone substitute regeneration often occurs with an autograft due to unpredictable resorption rates^4^. Most of all, there is a limited supply of autograft bone available for clinical use. Current bone grafting materials may be used to solve this challenging problem, though they are limited in regard to large defect repair.

Tissue engineering strategies have shown that the insertion of scaffolds into the skeletal defect site can enhance bone regeneration^5,6^. These tissue engineered scaffolds are able to provide an environment for the seeded cells in which normal regeneration can occur. The ideal bone scaffold is mechanically and biologically compatible, does not elicit an immune response, and has an absorption rate similar to the bone formation rate. Various types of biomaterials have been used as bone grafts, including polymers^7–9^, ceramics^10,11^ and composite materials^12–14^. Despite intensive research within bone tissue engineering, the properties of the scaffolds are still far from those of natural cortical bone^15^. No currently available bone scaffolding has been proven to possess the level of osteogenic factors to overcome the normal regenerative limit nor have they satisfactorily met the mechanical strength necessarily to be comparable to natural cortical bone. Due to the biochemical and biomechanical similarity with the natural bone, collagen hydroxyapatite composites are desired materials in bone tissue engineering and are currently widely evolved as a composite bone graft material prepared by a self-organization mechanism or the crosslinking agent, glutaraldehyde^16,17^. Recently, there was an attempt to use an electrospun collagen and hydroxyapatite nanocomposite scaffold for bone regeneration^18,19^, but weak mechanical strength still became a major issue. Frequently, hydrophobicity of collagen hydroxyapatite scaffold can result in poor cellular attachment, proliferation, and a high apoptotic rate for osteogenic cells^20^.

Rapidly evolving technology has enabled the development of biomimetic composite biomaterials at the nanoscale to achieve an improved bone scaffold^21^. Recently, we developed a polydopamine-laced hydroxyapatite collagen calcium silicate (HCCS-PDA) biomaterial to repair a critical-sized defect (CSD) in bone. Incorporating dopamine (DA) into the collagen hydroxyapatite material yields a novel group of biomaterials with superb mechanical strength as compared to many commercial bioceramics^22^. In addition, it has been noted that DA has stimulated proliferation, attachment, and differentiation on osteoblasts through the DA receptors^23^.

For many years, the introduction of numerous bone scaffold materials has demonstrated that utilization of mesenchymal stem cells (MSCs) and scaffolding materials can facilitate bone regeneration^24–27^. Previous studies have used single MSCs, though recent studies have shown that MSC aggregates can facilitate improved *in vitro* osteogenic differentiation and *in vivo* bone formation compared to the monolayer culture in terms of higher viability, antigenicity, and anti-inflammatory properties. These characteristics enable the MSC aggregates to enhance cell survival after implantation^28^, which makes rat mesenchymal stem cell (rMSC) aggregates a more favorable tissue engineering preclinical model for delivery into the grafting material to achieve enhanced bone regeneration.

In this study, we used our in-house HCCS-PDA and HCCS in particulate form to assess the capability of these bone grafting biomaterials to stimulate bone regeneration. Emphasis was placed on how DA incorporation into the HCCS biomaterial affects rMSC proliferation, differentiation, and physical properties *in vitro* and *in vivo* bone formation to repair the rat calvarial critical sized defect (CSD).

## Materials and Methods

### Material Preparation and Analysis

#### Dopamine Laced Hydroxyapatite Collagen Calcium Silicate (HCCS-PDA)

Hydroxyapatite and collagen (HC) slurry was biomimetically synthesized by the co-precipitation method using *in situ* hybridization of calcium silicates (CS) with HC powder^29^. The powders of HC (150 mg), dopamine (5 mg) and calcium hydroxide (100 mg) were mixed and cross-linked with 383 µL of enTMOS (bis [3-(trimethoxysilyl)-propyl] ethylenediamine) before adding APS (80 µL) and PBS. As the mixture began to thicken, it was transferred into 9 mm inner diameter disk molds and pressed with Teflon-covered glass on both sides to smooth the surface. The structures were dried for 7 days, broken into particulates larger than 2 mm in their longest dimension selected for, then sterilized with cold ethylene oxide (EO) gas before use.

To examine the cell-to-material interaction *in vitro*, 1 mL of HC slurry was washed with 1 mL of 200-proof methanol and then centrifuged (5,000 rpm). This was repeated three times to remove excessive moisture, then 1 mL fresh methanol was added. After adding 0.0296 g of Ca(OH)_2_ and 44.4 μL of 95% enTMOS, the tube was vortexed for 15 seconds and then sonicated in Ultrasonic Cleaner FS20 (Fisher Scientific) for 15 minutes. The mixture (50 μL) was coated on each 35 mm dish using P-6000 Spin Coater (Special Coating System Inc., Indianapolis, IN, USA) while being centrifuged at 6,000 rpm for 20 seconds. For preparation of the HCCS-DA coating, an additional 0.011 g of dopamine and 44.4 μL of ammonium persulfate (2.5%) were added. Coated dishes were washed with distilled water (dH_2_O) and sterilized under UV overnight before use.

#### Scanning Electron Microscopy (SEM) and Energy Dispersive X-ray Spectroscopy (EDX) Analysis

Cross-sectioned HCCS-PDA, HCCS, and calvaria particles were fixed on a holder, sputter-coated with platinum (4 nm) in a vacuum and imaged using a Hitachi S-4700 Cold Cathode Field Emission SEM (Hitachi High Technologies America, Inc., Schaumburg, IL USA). EDX analysis was conducted to determine the chemical elementary composition of the samples on three random cross-sectional regions using INCA operator software (Oxford Instruments Analytical, High Wycombe UK).

#### Contact Angle Measurement and Mechanical Testing

The contact angle where dH_2_O meets HCCS-PDA and HCCS surfaces was measured to determine the wettability of the HCCS-PDA and HCCS surface using a contact angle analyzer (CAM 200, KSV Instruments, Finland). Approximately 1 μL of water was dropped onto the surface of the HCCS-PDA and HCCS coated dishes and the contact angle was measured after a static time of 30 seconds. Three coated dishes from each material and five data points from each dish were used to calculate the mean and standard deviation.

The compressive strength of HCCS-PDA was determined using an Instron (model 4204, Canton, MA, USA) and compared to HCCS specimens. Samples were prepared in cylindrical shape with a 1:2 ratio of diameter (3.5 mm) to length (7.0 mm) using plastic molding and compressed by 0.5 mm/min uniaxial force, with five samples tested per group. The maximum strength value was measured from the stress-strain curve.

### Rat Mesenchymal Stem Cells from Bone Marrow

#### rMSC Isolation, Culturing, Characterization

The detail method of isolating, culturing and characterizing rMSCs by specific surface markers and Tri-linage differentiation were well described in the previous study^26^. Briefly, after the sacrifice of male Sprague-Dawley rats (300 g, 12 weeks old, Charles River, Wilmington, MA USA), femurs were dissected with both ends cut to flush marrows with phosphate-buffered saline (PBS). The bone marrow cells were cultured in growth media (Dulbecco’s modified Eagle’s medium (DMEM) containing 10% fetal bovine serum (FBS), 1x GlutaMax (Thermo Fisher Scientific Inc., Rockford, IL, USA), and 1% penicillin/streptomycin (Thermo Fisher Scientific Inc., Rockford, IL, USA). After 24 hours, non-adherent cells were removed and cells were subsequently passaged using 0.05% trypsin-EDTA (Thermo Fisher Scientific Inc., Rockford, IL, USA) with fresh medium supplied every 3 days during expansion. Animal protocol was approved by the Institutional Animal Care and Use Committee (IACUC) at the University of North Carolina (UNC) at Chapel Hill (Approved protocol number 15–273) and all experiments were followed the guidelines by the IACUC at the UNC.

#### rMSC Aggregates for *In Vivo* Implantation

rMSCs (2 × 10^3^ cells) in suspension were seeded in 96-well rounded bottom, ultralow attachment plates (Costar, Corning Inc. Life Sciences, Lowell, MA USA) and aggregated via the forced aggregation method. Briefly, rMSCs were centrifuged at 1,400 rpm for 4 minutes to form aggregates. Aggregates were grown in growth media for 3 days and differentiated into osteogenic cells by suppling fresh osteogenic media every other day for 14 days. Aggregates were imaged using Nikon Eclipse T*i*-U inverted microscope and the diameter of each aggregate was measured using NIS-Elements Basic Research imaging software (Nikon Instruments Inc., Melville, NY USA).

For Collagen type I immunostaining, aggregates were embedded in optimal cutting temperature (OCT, Sakura Japan) solution and sectioned at 5 µm using a cryostat, fixed with 4% PFA, rinsed, and incubated for 30 minutes with 3% H_2_O_2_ solution. After blocking for 30 minutes with 5% FBS in PBS–Triton solution, aggregates were incubated overnight at 4 °C with rabbit primary antibody against rat collagen type I (NB600-408, Novus Biologicals, LLC, Littleton CO USA). After three rinses, sections were incubated with secondary biotinylated goat anti-rabbit IgG antibody (NB730-B, Novus Biologicals, LLC, Littleton CO USA) for 30 min at RT. After incubation in ABC complex (Vector Laboratories, Burlingame, CA USA), DAB Chromogen solution (Dako, Santa Clara, CA USA) was added for 5 to 20 minutes until a brown color developed. For Alizarin Red staining, sections were incubated with 1% Alizarin Red Solution (pH 4.2) for 10 minutes at RT, then washed with dH_2_O. The sections were dehydrated with 95% and 100% ethanol, immersed in xylene solution, and then mounted with coverslip using Cytoseal (Richard-Allan Scientific Co, San Diego, CA USA). Quantitative analysis for mineral formation and collagen synthesis over time was performed using Image J software (U. S. National Institutes of Health, Bethesda, MD USA) after image acquisition.

### *In Vitro* Analysis of HCCS-PDA and HCCS by rMSCs

#### Live/Dead Assay

After culturing rMSCs on a 35 mm dish coated with HCCS-PDA and HCCS for 3 days, cell viability was examined with the Live/Dead Assay Kit (Molecular Probes, Life Technologies Corporation, Grand Island, NY USA) via company instructions. Viability was assessed with a Nikon Eclipse T*i*-U inverted microscope (Nikon Instruments Inc., Melville, NY USA) by quantifying live and dead cells, detected by Calcein as green fluorescence and Ethidium homodimer-1 (EthD-1) as red fluorescence.

#### Cellular Proliferation, Attachment, and Mineralization

A total of 50,000 rMSCs per well were plated in HCCS-PDA and HCCS coated dishes to assess the rMSC proliferation using the MTS Cell Proliferation Assay Kit (Promega Co., Madison, WI, USA) following company instructions. The MTS assay (3-(4, 5-dimethylthiazol-2-yl)-5-(3 carboxymethoxyphenyl)-2-(4-Sulfophenyl)-2H-tetrazolium) reacted with cells at 37 °C for 1 hour. After transferring the solution into a 96 well plate, growth was measured on days 1, 3, 5 and 7 at 490 nm using a microplate reader (Biorad, Hercules, CA, USA). To detect rMSC proliferation on HCCS-PDA or HCCS coated culture dishes, as well as on a culture dish control, 5-bromo-2′-deoxyuridine (BrdU: Life Technologies, Grand Island, NY, USA) was incorporated with rMSCs for 2 hours and detected using anti-BrdU antibody (Santa Cruz biotech, CA) by following the company’s protocol.

The rMSCs were seeded in HCCS-PDA and HCCS coated dishes at a density of 50,000 cells per well and incubated at 37 °C. At 2, 6, 16, and 24 hours, Number of attached cells was counted from the microscopic images acquired (n = 5 each group).

Mineral nodule formation was examined by directly treating rMSCs with DA and culturing rMSCs on coated dishes under osteogenic media. To measure and compare bone mineralization capabilities, 100,000 cells were seeded per well in 12-well dishes. Fresh osteogenic media was supplied every 3 days for 28 days. Cells were harvested on day 10, 14, 21, and 28, fixed in 95% ethanol for 30 minutes at RT, washed with PBS, and stained with 1% Alizarin Red (pH 4.2) for 10 min at RT. Quantitative analysis was performed by elution with 1% CPC for 30 minutes at RT and OD was measured at 570 nm.

The rMSCs were pretreated with the DA receptor antagonists SCH23390 (Tocris Bioscience, Bristol, UK), Eticlopride (Tocris Bioscience, Bristol, UK), GR103691 (Tocris Bioscience, Bristol, UK), and L741742 (Tocris Bioscience, Bristol, UK) with final concentrations of 1 mM, 1 mM, 0.5 mM and 1 mM, respectively. After 30 minutes, DA (50 µM) was added to the rMSCs. DA and agonists were treated after changing with fresh osteogenic media in every 3 days up to 14 days.

### CSD Calvaria Repair Using HCCS-PDA or HCCS Particles with or without rMSC Aggregates

#### Animal Calvaria Surgery

All animal surgical procedures were approved by IACUC at the University of North Carolina, Chapel Hill. Differentiated rMSCs aggregates in osteogenic media were incorporated with the HCCS-PDA and HCCS particulates. Six implantation groups were used (HCCS-PDA only, HCCS-PDA + rMSCs, HCCS only, HCCS + rMSCs, calvaria autograft control, and empty defect control) with 4 rats per group, for a total of 24 male Sprague-Dawley rats. An 8 mm CSD was created after anesthetization by Ketamine-HCl injection (10 mg/kg: Puteney Inc., Portland, ME USA). The surgical procedure and the fluorochrome injections for the MAR have been well described in the previous study^30^.

#### Micro-CT Analysis

The harvested calvaria were scanned with a Skyscan micro-CT (Skyscan 1076; Skyscan, Aartselaar, Belgium) to acquire images at 40 kV, 1000 mA with a 720 ms integration time. Acquired images were reconstructed as 3D image using ITK-SNAP software. Following the reconstruction process, radiodensity was measured using Geomagic Design X™ (3D Systems Inc., SC USA) software in the CSD site from three animals in each group. Animals with autografts served as the positive control. The total radiodensity in the defect area was represented in mm^3^.

#### Fluorescence Microscopy for Mineral Apposition Rate (MAR)

The detailed method for non-decalcified slide preparation was described in our previous study^28^. The fluorescent images of completed sections were acquired by using a fluorescence microscope with 10X magnification of TRITC and FITC filters, and the each images were stitched together using NIS-Elements Basic Research imaging software.

#### New Bone Formation (NBF) Measurement

Calvaria explants from each group were stained using Stevenel’s Blue and counterstained with Van Gieson to visualize newly formed bone (NFB) tissue. Colored microscopic images of the medial (central) sagittal histologic sections were acquired under 10x magnification using Nikon Eclipse Ti-U camera with automatic stage, then merged using Nikon software to stich each image as one figure. The newly formed bone area (B.Ar.) and the total defect area (T.Ar.) were quantified using Image J software to calculate the NFB in % followed by the standardized protocol^30^.

### Statistical Analysis

All quantitative results were represented as mean ± standard deviation. When the *p* value was less than 0.05, the differences were considered to be statistically significant (*p < 0.05). While comparisons between two groups were made with paired t-tests, comparisons within groups were made by ANOVA.

## Results

### Gross and Microscopic Images of HCCS-PDA, PDA, and Calvaria

**Figure 1:**
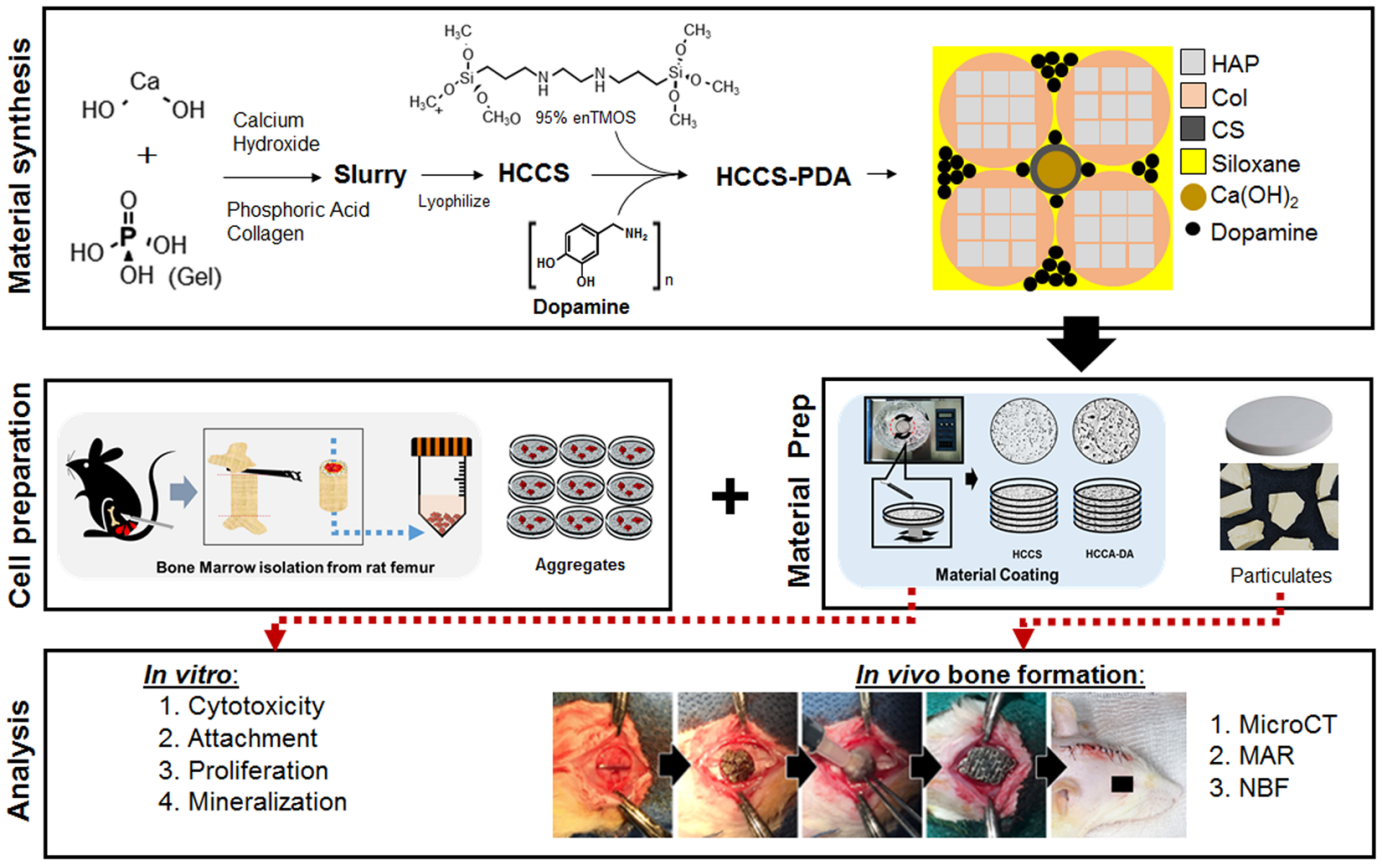
Schematic of the study.

**Figure 2:**
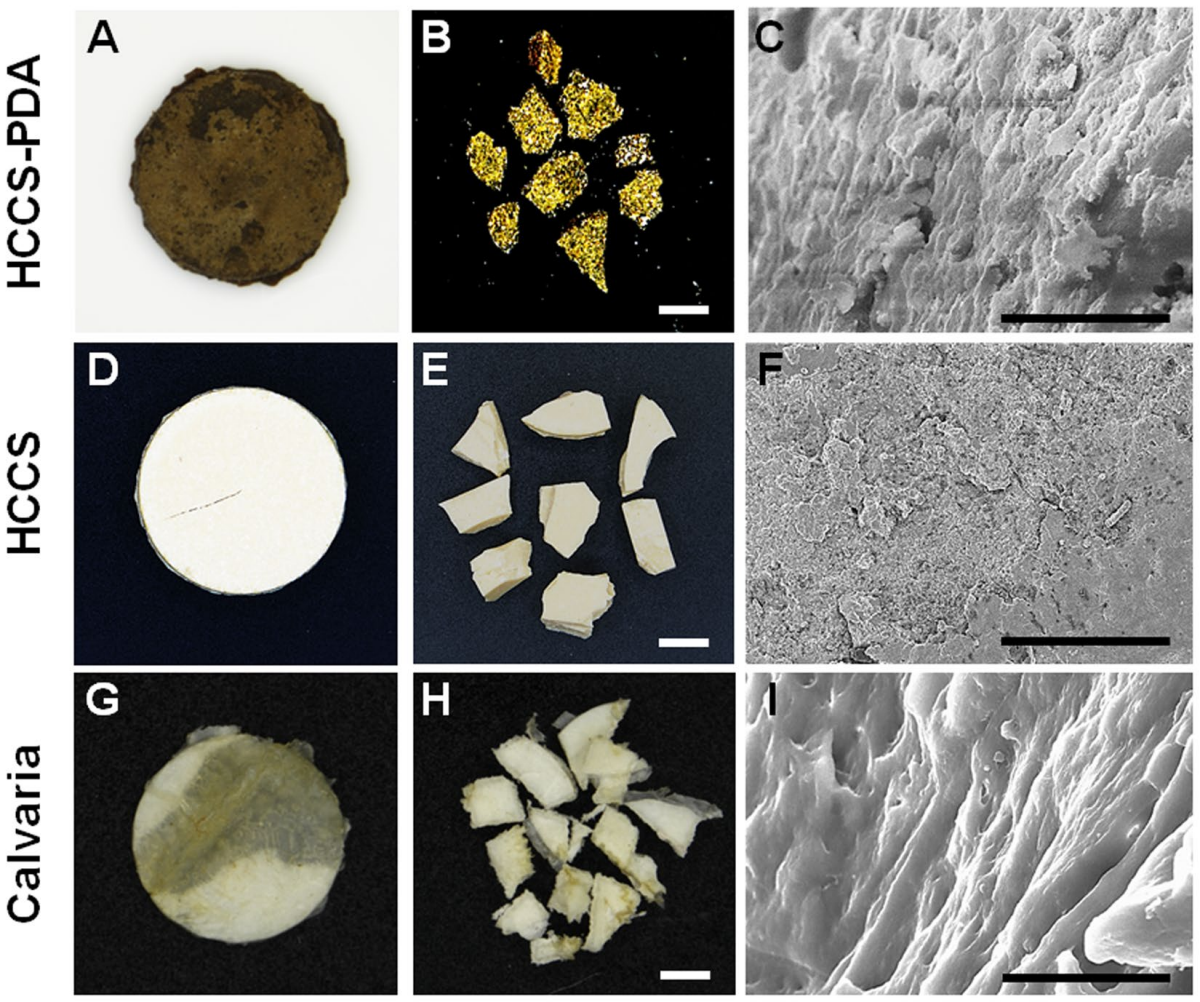
Representative gross images of HCCS-PDA, HCCS, and calvaria disk samples (**A**,**D**,**G**) and particulate forms (**B**,**E**,**H**), scale: 2500 μm. Scanning electron microscopy images of each particulate’s cross-sectional view (**C**,**F**,**I**), scale: 10 μm. The disk dimensions are 16.5 mm in diameter and 2 mm in thickness.

HCCS-PDA and HCCS were successfully fabricated using the co-precipitation method (Fig. 1). The dimension of the fabricated disks was 2 mm in thickness and 16.5 mm in diameter (Fig. 2A and D). SEM images were acquired of the particles prepared in this study (Fig. 2C,F and I), with the cross-sectional view of HCCS-PDA demonstrating a lamellar structure (Fig. 2C) similar in appearance to the calvaria (Fig. 2I). This similar morphology is due to the polydopamine cross-linkage, which provide a long range molecular order. HCCS particles without DA, however, showed fine granular surface in the cross-sectional view (Fig. 2F), which implies brittle characteristics of the material.

### Mechanical, Atomic and Surface Property Assessment for HCCS-PDA

**Figure 3:**
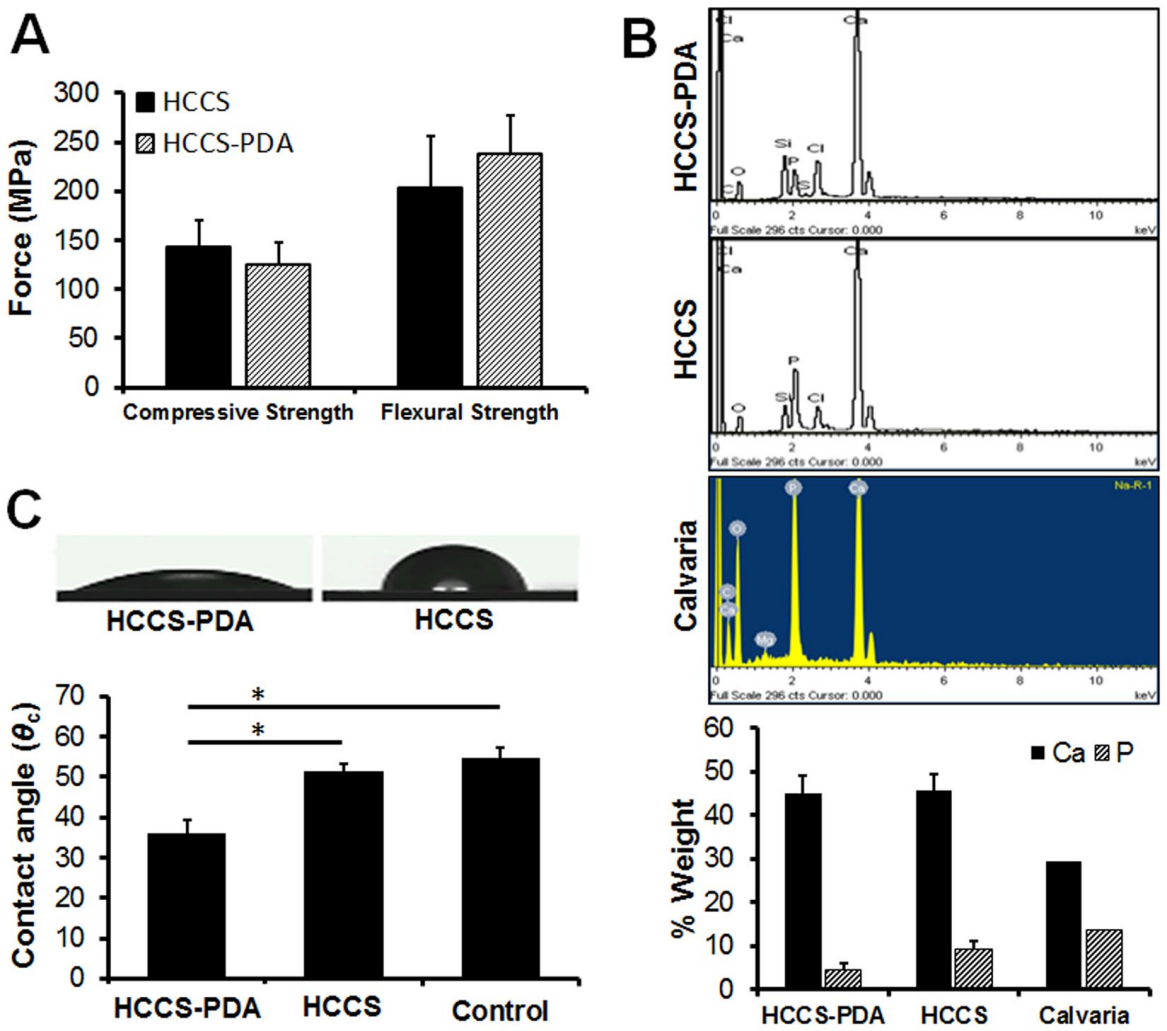
The compressive and flexural strength of HCCS-PDA and HCCS was also measured (**A**). EDX analysis for calcium and phosphate composition on three randomly selected areas after acquiring SEM images of HCCS-PDA, HCCS, and calvaria (**B**). The water contact angle was measured on the surface of a culture dish coated with HCCS-PDA or HCCS, with the control of a culture dish without coating (**C**).

The mean values of the compressive strength were 124.36 ± 22.89 and 143.11 ± 27.27 MPa for HCCS-PDA and HCCS, respectively. Through there is no significant difference in compressive strength, the flexural strength of HCCS-PDA was higher than that of HCCS. Certainly, DA increased biaxial flexural strength of HCCS from 203.25 ± 53.14 to 237.44 ± 39.77 MPa (Fig. 3A). Water contact angles in the HCCS-PDA group (35.90 ± 3.51°) were lower than that in HCCS group (51.43 ± 1.79°), indicating that the HCCS-PDA surface has greater hydrophilicity than the HCCS surface (Fig. 3C). The EDX spectra are shown in Fig. 3B along with the weight percentages of mineral elements of HCCS-PDA and HCCS. The total percentage of Ca in both HCCS-PDA (44.9%) and HCCS (45.64%) was higher than that in the calvaria autograft (29.35%), due to additional calcium silicate, and the percentage of P (4.56% in HCCS-PDA and 9.27% in HCCS) was lower than that in calvaria (13.71%).

### Isolation and Characterization of Bone Marrow Rat Mesenchymal Stem Cells (rMSCs)

**Figure 4:**
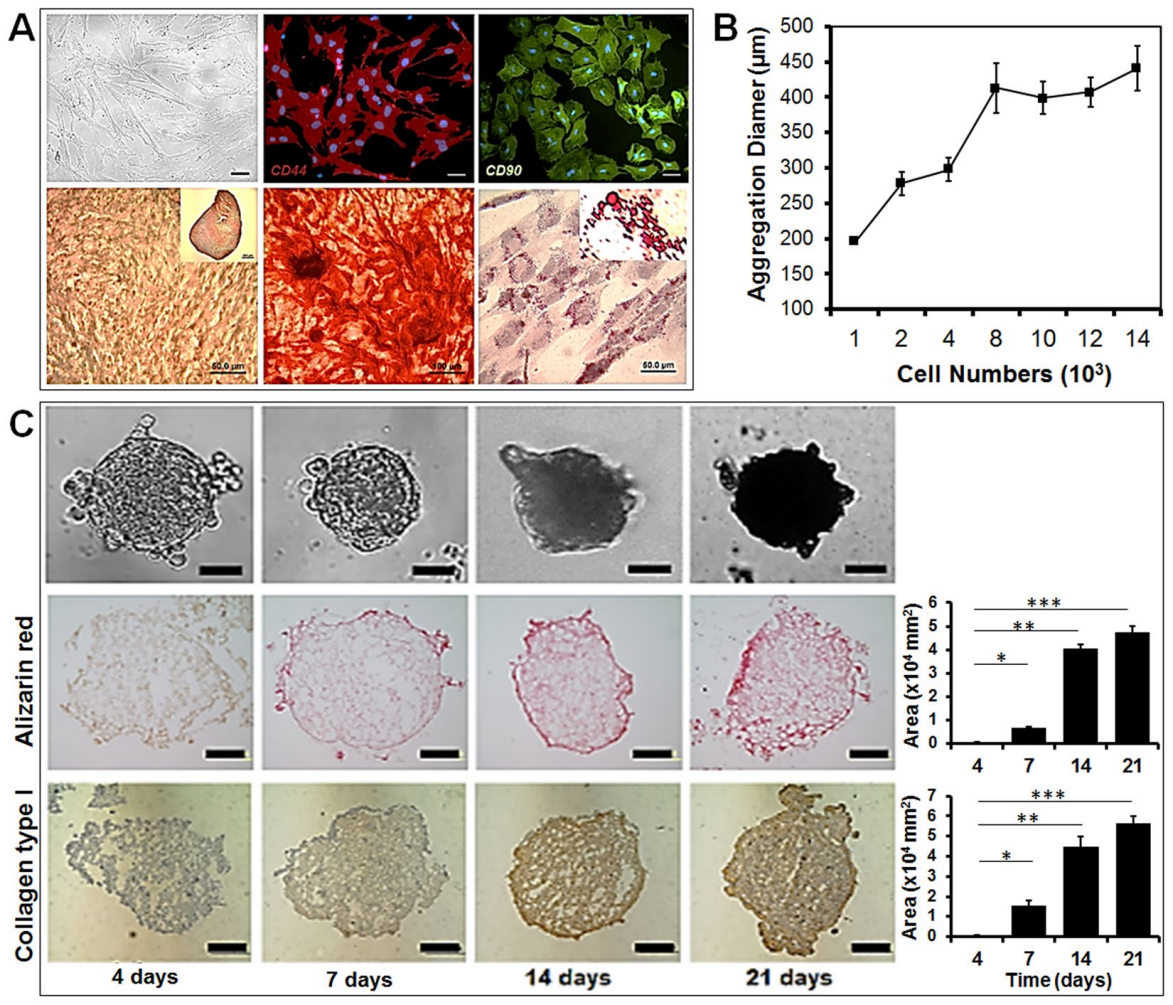
Characterization of rat mesenchymal stem cells (rMSCs) from bone marrow with DIC microscopic picture; rMSCs stained with CD44 and CD90 antibody conjugated with TRITC and FITC; tri-lineage differentiation capability into chondrogenic lineage after 3D aggregation culture for 21 day, osteogenic lineage after 21 days, and adipogenic lineage after 21 days (scale bar: 50 μm) (**A**). The rMSC aggregate diameter based on cell number, with approximately 2,000 rMSCs used to form aggregates with diameters between 250 and 300 μm (**B**). Characterization of osteogenic differentiation of rMSC aggregates over 21 day, with 3D aggregates analyzed by immunostaining with collagen type I antibodies for collagen synthesis and by Alizarin Red S staining for mineralization (**C**). Image J software was used to quantify the positively stained area (scale bar: 50 μm).

The method of bone marrow MSCs isolation from Sprague Dawley rats has been well described in the previous study^29^. Primary cultured rMSC at passage 4 were characterized for their phenotype determined by DIC imaging. Colony forming capability was assessed by single MSCs. Antibodies against rMSC specific surface markers, CD44 and CD90 (Fig. 4A) confirmed positive and negative expression for CD45 and CD34 (data not shown) antibodies.

Chondrogenic media induced differentiation of rMSC 3D pallet culture after 21 days. Safranin O staining was positive for the neocartilage-like matrix produced and lacuna were detected in the matrix (Fig. 4A). Osteogenic media induced evident mineral formation on rMSCs after 21 days and the mineral nodules binding to calcium were clearly visualized by Alizarin Red S staining (Fig. 4A). The lipid droplets were clearly visualized by Oil Red O staining in rMSC after 21 days of using adipogenic induction medium (Fig. 4A).

We observed that rMSC aggregates clearly express Col I protein and displayed mineral formation during osteogenic differentiation up to 21 days. Both collagen type I and mineralization of rMSCs aggregates increased over time, up to 21 days (Fig. 4C), with initial visibility 7 days after differentiation. This *in vitro* osteogenic differentiation indicated rMSCs in 3D aggregate form could differentiate into osteogenic cells by secreting natural bone-like extracellular matrix to mimic the natural 3D environment of the bone.

### Effects of HCCS-PDA on Viability, Attachment, Proliferation and Mineralization

**Figure 5:**
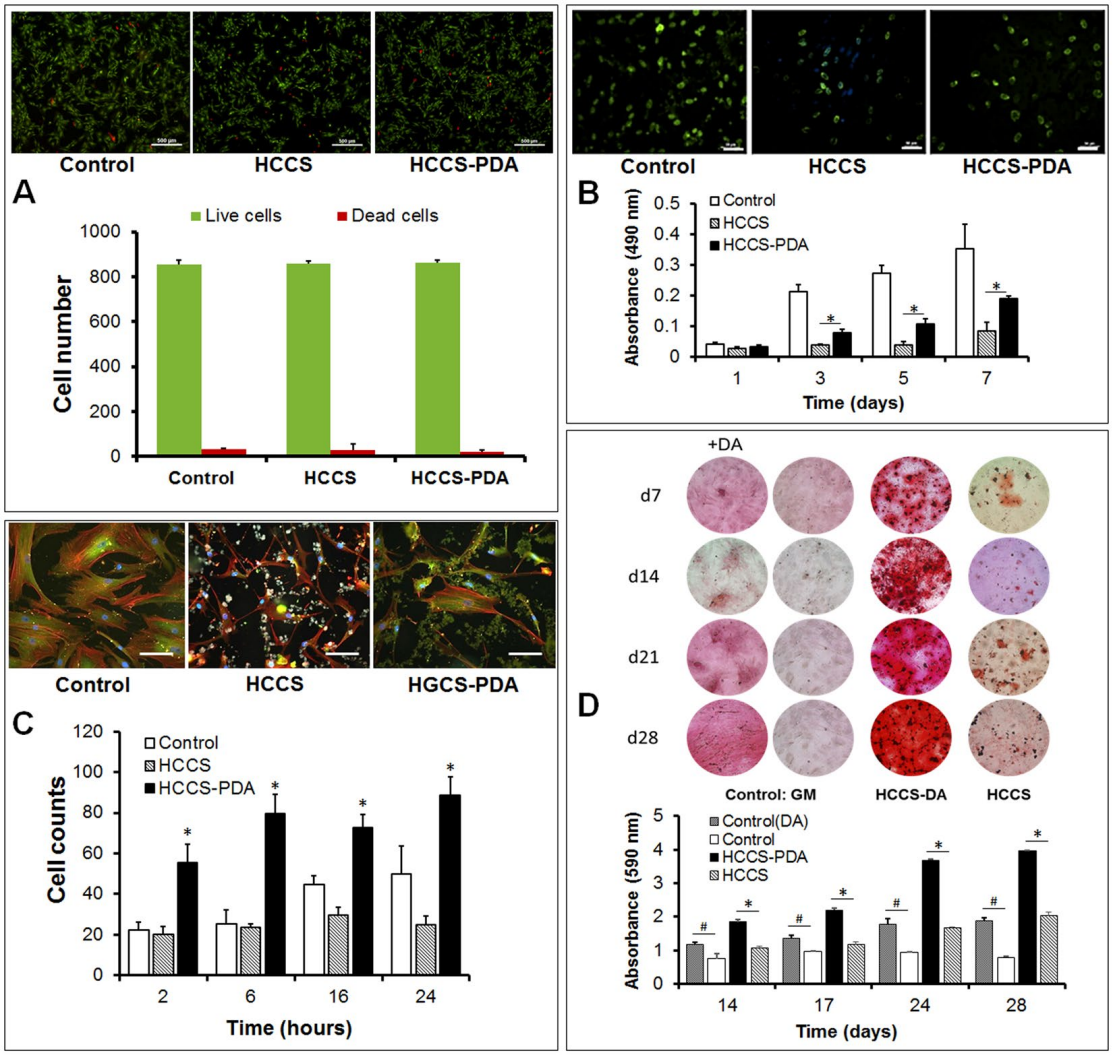
Live/Dead Assay was done to test the toxicity of HCCS-PDA and HCCS, with viable rMSCs staining as green by Calcein-AM and dead rMSCs staining as red by EtD-1 (**A**), scale: 500 μm. The rMSC proliferation on the HCCS-PDA and HCCS coated dishes was assessed by MTS on 1, 3, 5, and 7 days of culturing, with a non-coated culture dish as the control (**B**), scale: 50 μm. rMSC adhesion assay on the coated culture dish with HCCS-PDA and HCCS was performed after 2, 6, 16 and 24 hours by counting the number of attached cells. Cell morphology was observed after immunostaining with focal adhesion assay kit (**C**), scale: 10 μm. Mineral nodule formation after Alizarin Red S staining and CPC quantification was assessed. Osteogenic differentiation was performed using 50% reduced osteogenic factors in the media. Enhanced mineralization was observed in the rMSCs cultured with growth media for 28 days on HCCS-PDA, with the formation of small minerals evident (**D**), GM: growth media and OM: osteogenic media. Extracted solution by 1% CPC was measured at an absorbance of 570 nm (n = 5, *p < 0.05 vs. in osteogenic media on HCCS, and ^#^ *p* < 0.05 between in growth media without DA and with DA). Image J analysis of the mineral coverage of the culture dish. Both CPC and Image J analysis yielded similar results.

First, we evaluated the cytocompatibility of rMSCs cultured with HCCS-PDA and HCCS, compared to the culture dish as control (887 ±  × 34 live cells and 12 ± 2 dead cells). The Live/Dead staining with Calcein-AM (green) and ethidium homodimer (red) showed 864 ± 21 live cells and 21 ± 3 dead cells in the rMSC culture on HCCS-PDA, while the culture on HCCS showed 859 ± 11 live cells and 29 ± 3 dead cells (Fig. 5C). The adhered cell morphology of rMSCs cultured on HCCS-PDA, HCCS and culture dish at 1 day was observed using actin filaments (red), vinculin (green), and nuclei (blue) fluorescent staining. As shown in Fig. 5A, rMSCs on the HCCS-PDA (7,537 ± 3,769 pixels) exhibited greater spreading with a better cytoskeleton than on HCCS (2,013 ± 708 pixels), though less spreading in comparison to the control (11,697 ± 8637 pixels, in-house data). The adhesion assay results revealed that the number of attached cells cultured on HCCS-PDA (55 ± 9 cells at 2 h, 80 ± 9 cells at 6 h, 73 ± 6 cells at 16 h, and 89 ± 9 cells at 24 h) were significantly higher than those on the HCCS (20 ± 3 cells at 2 h, 23 ± 2 cells at 6 h, 29 ± 4 cells at 16 h, and 25 ± 4 cells at 24 h and control (22 ± 4 cells at 2 h, 25 ± 6 cells at 6 h, 45 ± 4 cells at 16 h, and 50 ± 14 cells at 24 h). To determine rMSC growth, the MTS assay was performed on days 1, 3, 5, and 7. OD measurements of the HCCS-PDA group were 0.03 ± 0.01 on day 1, 0.08 ± 0.01 on day 3, 0.11 ± 0.02 on day 5, and 0.19 ± 0.01 on day7, while the HCCS group had 0.03 ± 0.01 on day 1, 0.04 ± 0.01 on day 3, 0.04 ± 0.01 on day 5, and 0.08 ± 0.03 on day7. The rMSCs on HCCS-PDA displayed a greater growth rate up to day 7, with significant difference in the proliferation rate (*p < 0.05), except on day 1(*p > 0.05). Short-term growth results indicated that rMSC proliferation was greater with HCCS-PDA than HCCS. Figure 5B represents proliferating rMSCs labeled with BrdU on day 3. The rMSCs on HCCS-PDA (37 ± 5 cells per 0.15 mm^2^) showed a higher number of proliferating cells than those on HCCS (28 ± 7 cells per 0.15 mm^2^ cells) though the control (culture dish) showed highest number of proliferating cells (70 ± 5 cells per 0.15 mm^2^).

Figure 5D shows the microscopic images of the Alizarin Red S staining for rMSCs osteogenically differentiated on HCCS-PDA, HCCS, and control (culture dish) for 7, 14, 21, and 28 days. At day 7, the Alizarin Red S staining showed slight reddish dots on both HCCS-PDA and HCCS group, while the control group indicated almost no positive staining. Slightly enhanced staining was observed on day 28 for the control group with PDA, while the HCCS-PDA group showed more intense staining compared to the HCCS and control without PDA. This indicates that PDA can facilitate the mineralization of rMSCs. HCCS-PDA and HCCS without cells were used as the negative controls and did not form any mineral nodules (figure not shown).

**Figure 6:**
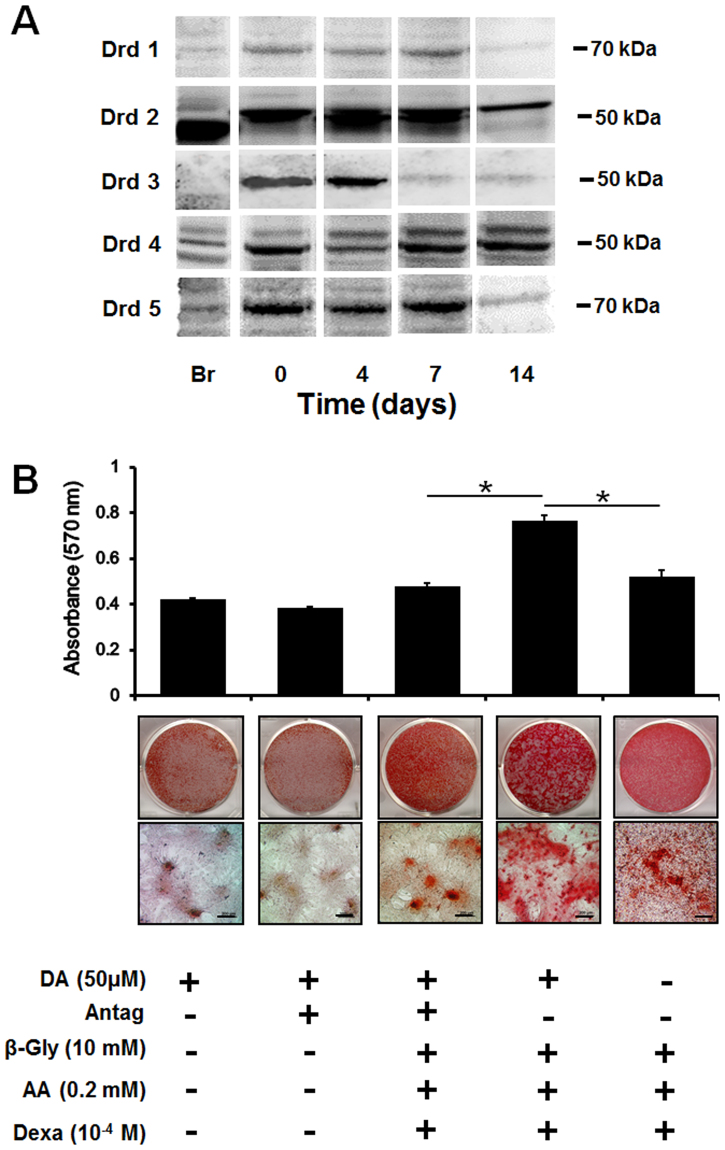
Western Blot analysis of DA receptors expressed on the rMSCs during osteogenic differentiation on days 0, 4, 7, and 14 (**A**). Each loading was normalized by beta actin (not shown here). The relative molecular weights of each DA receptor were labelled on the right side of the blot image and mouse brain was used as the control (denoted as Br). The receptor mediated DA effect on mineralization by rMSCs pretreated with/without DA antagonists (SCH23390, Eticlopride, GR103691, and L741742), as evaluated with Alizarin red S staining to detect mineral nodule formation (**B**), scale: 200 μm. The control was DA treated with/without antagonists in growth media (n = 3 per group; *p < 0.05 compared with DA treated with antagonists pretreated group at same time point). DA: dopamine, Antag: antagonists, *β*-Gly: *β*-Glycerphosphate, AA: ascorbic acid, and Dexa: dexamethasone.

To confirm the dopaminergic effect on enhancing mineralization though dopamine receptors, we verified the presence of dopamine receptors in rMSCs and observed whether the receptors continued to be expressed during osteogenic differentiation. In this regard, D2 and D4 receptors were clearly expressed at day 14 of osteogenic differentiation (Fig. 6A). We pre-treated cells with a dopamine antagonists prior to dopamine addition to determine whether mineralization is also enhanced in rMSCs cells. Our results indicated that there was significant reduction of mineralization in rMSCs with DA antagonist treatment in osteogenic media at 14 days, whereas no changes were evident in the culture media at 14 day (Fig. 6B).

### Micro-CT and Histomorphometric Analysis

**Figure 7:**
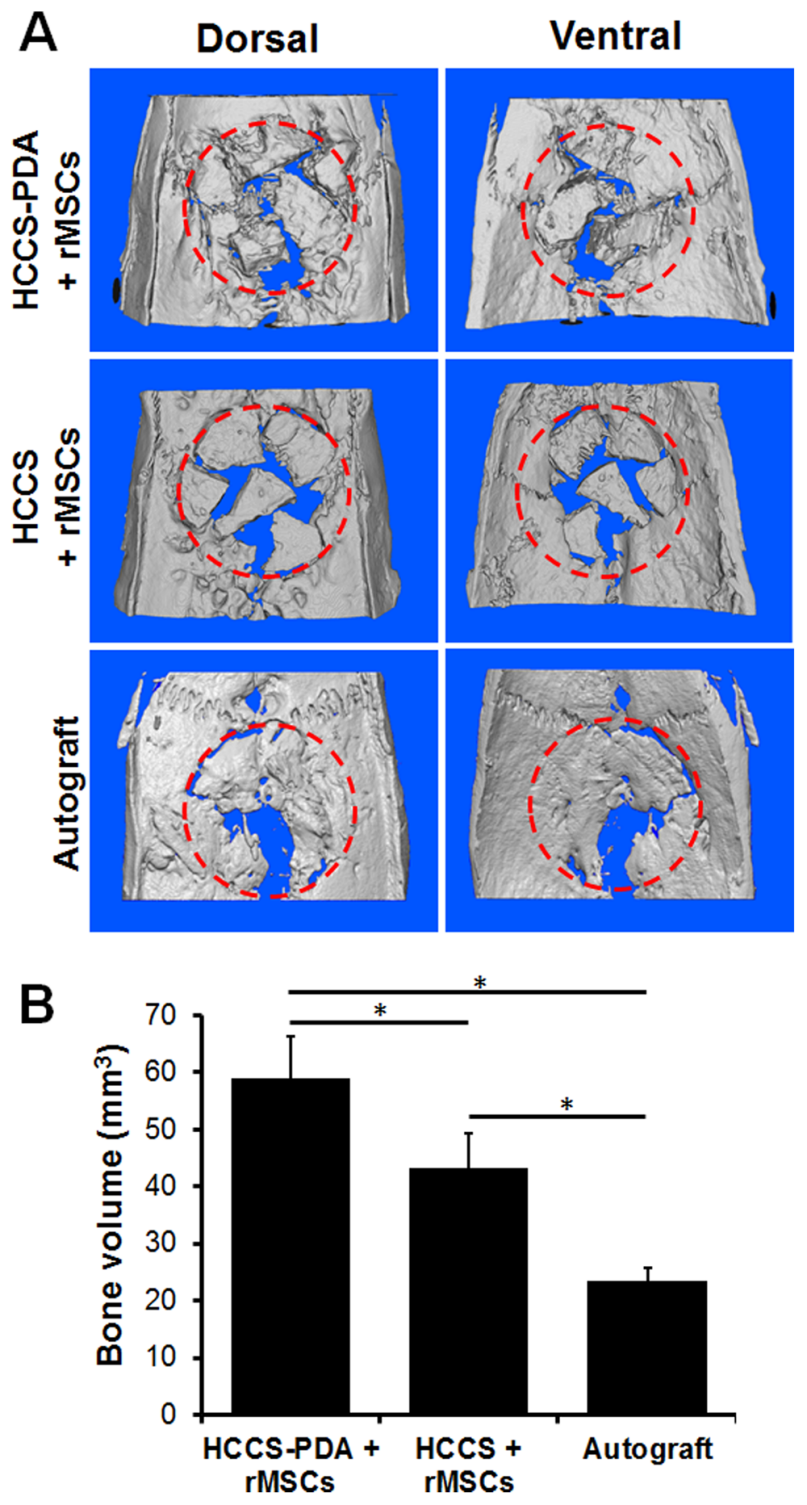
Micro-CT images of explanted critical sized calvarial defect after 12 weeks of implantation with HCCS-PDA with rMSC aggregates, HCCS with rMSC aggregates, and autograft (**A**). Red dotted circle: defect site (red dotted circle: 8 mm in diameter). Bone volume of the defect site including HCCS-PDA/HCCS and newly formed bone was quantified (*n* = 4, *p < 0.05) (**B**).

After 12 weeks post-implantation, the entire calvaria was explanted and underwent fixation. Micro-CT analysis revealed that new tissues grew into the CSD with particles evident in both HCCS-PDA and HCCS groups. The HCCS-PDA group seeded with rMSC aggregates led to the formation of bony tissue in most of the defect was evident after 12 weeks. In contrast, the groups of HCCS seeded with rMSC aggregates or HCCS-PDA without rMSC aggregates failed to bridge the CSD (Fig. 7A). The newly formed bone volume of the HCCS-PDA with rMSC aggregates group (59.01 ± 7.21 mm^3^) was higher than the HCCS with rMSC aggregates group (43.22 ± 5.99 mm^3^). Figure 7A indicated that the majority of the particles merged with the surrounding host bone rather than to the center of the defect. Analysis of the autograft groups by measuring bone radiodensity using Geomagic Design X™ software indicated 23.35 ± 2.48 mm^3^ (Fig. 7A). Overall, the HCCS-PDA particles with rMSC aggregates showed superior bone regeneration in the defect site compared to the other groups.

**Figure 8:**
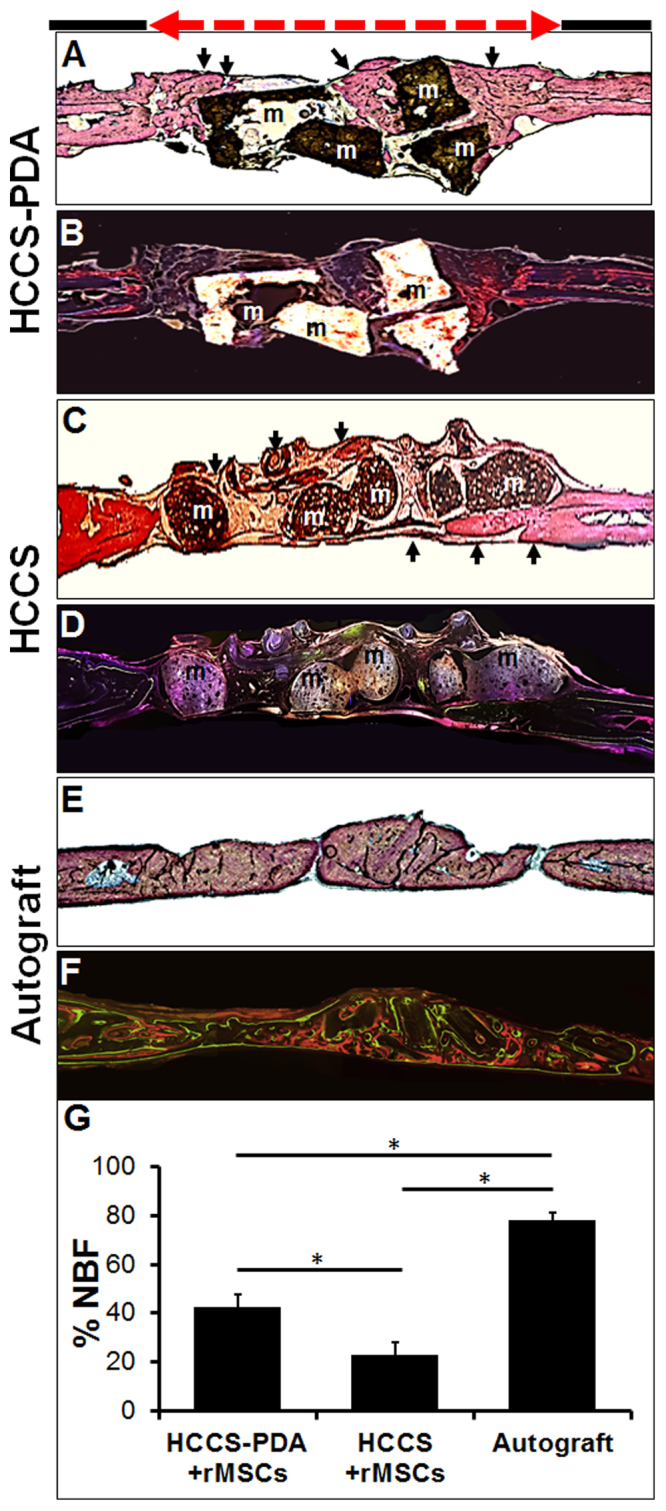
Histological and fluorescent medial-sagittal sections of the defect area after 12 weeks of implantation with HCCS-PDA with rMSC aggregates (**A** and **B**), HCCS with rMSC aggregates (**C** and **D**), and autograft (**E** and **F**). Stevenel Blue and Van Gieson staining revealed new bone formation (NBF), represented by light reddish color (arrows). The area was quantified as a percentage using Image J software, *p < 0.05 (**G**). Fluorescence images labeled with calcein and tetracycline dye were acquired before performing Stevenel Blue and Van Gieson staining. The defect site (red dotted line) is 8 mm.

Following sample staining with Stevenel’s Blue and counter-staining with Van Gieson, histological analysis demonstrated that the HCCS-PDA with rMSCs group had new bone filling which partially bridged the center of the defect at 12 weeks post-implantation (Fig. 8A). The NBF around the particles was well integrated at the interface between the HCCS-PDA and newly formed bone in the defect. In the HCCS only group, NBF was less visible versus the HCCS-PDA with rMSCs group, as evident at the peripheral site of the defect (Fig. 8B). There was an absence of NBF in the center of defect, with only intervening fibrous tissue evident. Nearly full regeneration was displayed in the defect area with use of autografts (Fig. 8C). In the Fig. 8G, quantitative measurements of NBF calculations were 42.73 ± 4.83% for the HCCS-PDA with rMSCs group, 22.99 ± 5.24% for the HCCS with rMSCs group and 77.9 ± 3.09% for the autograft, respectively. HCCS-PDA and HCCS groups without cells have 27.31 ± 6.84% and 23.82 ± 4.04%, respectively (data not shown here).

## Discussion

With inspiration drawn from the natural bone matrix, the collagen modified siloxane–calcium silicate (HCCS) nanocomposite scaffold is a hydroxyapatite and collagen derivative developed to mimic the physical and chemical properties of natural bone. Collagen can improve the scaffold mechanical strength profile, though the weak bonding between hydroxyapatite-collagen (HC) particles and siloxane is prone to brittle fracture and subsequently results in a relatively low compressive strength as compared to natural bone. The application of calcium silicate (CS) and dopamine (DA) to HC led to the creation of a new scaffolding material, HCCS-PDA, to overcome this problem. Addition of DA not only reinforced the mechanical strength of the material, but also improved osteoblast adhesion, proliferation, and differentiation upon DA release, indicating HCCS-PDA to be an ideal scaffolding candidate for bone regeneration. This study was designed to explore the interaction of HCCS-PDA with rMSCs through analyzing cytotoxicity, mechanical strength, cellular attachment/proliferation, and osteogenic differentiation *in vitro* and new bone formation under physiological conditions *in vivo*. To simplify the material preparation and solely focus on osteogenic property, the scaffold was implanted in particulate form *in vivo*.

Analysis of the acquired SEM cross-sectional views indicated that the HCCS-PDA sample surface bears more resemblance to calvaria than HCCS. In this regard, the laminated or fibrous structures were observed in both HCCS-PDA and calvaria. In contrast, clearcutting the HCCS surface indicated brittle characteristics under excessive force, resulting in lower flexural strength as compared to HCCS-PDA. Indeed, PDA seems to play a key structural role regarding the material. We also performed EDX analysis, indicating the Ca/P ratio of HCCS-PDA and HCCS somewhat differed to that of the calvaria control. The ratio between calcium and phosphate (Ca/P) is typically reported as 1.7 in natural bone. Because additional calcium source (Ca(OH)_2_) was added to the composite, HCCS-PDA and HCCS showed 9.8 and 4.9, which were far greater Ca/P ratios than that of natural bone (2.1) in this study. The level of both calcium and phosphate did not show any cytotoxicity after culturing with MSCs, offering an advantage in utilizing synthetic biomimetic biomaterials for bone regeneration. Introduction of a controlled amount of inorganic or organic components during material synthesis enables the provision of a chemically customized scaffold for those needing to regenerate bone with various amounts of inorganic compounds, though further in-depth studies are needed for future clinical applications.

HCCS is a nanocomposite made of collagenous hydroxyapatite with *in situ* pozzolanic formation of calcium silicate (CS) interacting among collagen, silica and calcium hydroxide (Ca(OH)_2_). Additional PDA was further crosslinked by ammonium persulfate to increase mechanical strength by 30%, close to the strength of cortical bone^30^. The results in Fig. 3C indicate that the compressive strength was not changed much after adding DA. However, the flexural strength of HCCS-PDA was higher than that of HCCS. DA could play a role in increasing this mechanical property of the HCCS scaffold by 34 MPa with minor effect (Fig. 3C). Although DA did not have much effect on mechanical strength of both HCCS-PDA and HCCS, it is known to polymerize well in wet environments such as the human body^31^ and expect to have more effects on cellular attachment, proliferation, and osteogenic differentiation.

The higher contact angle of HCCS-PDA versus HCCS indicates greater hydrophilicity caused by the DA group, as shown by previous biomaterial studies^32,33^. Amalgamated HCCS-PDA can leach DA monomers to the surrounding environment. This offers an advantage toward tissue engineering applications as MSC attachment, proliferation, and differentiation are enhanced on the surface of the porous, hydrophilic HCCS-PDA scaffold. However, the detailed mechanism underlying this enhanced cellular attachment on the PDA-enhanced surface is unclear, particularly whether the hydrophilic surface attract cells simply due to opposite charges or via chemical functional groups.

Thin layer coating with scaffolding material on culture dish using spin-coater is a useful cell culture tool to understand the interaction between cells and the coated material in association with cytotoxicity, attachment, proliferation and differentiation. Material cytotoxicity was tested by culturing MSCs on HCCS-PDA and HCCS-coated dishes via spin-coating and analyzing the results through use of the Live/Dead assay. The Live/Dead assay indicated that HCCS-PDA displayed minor toxic effects on rMSCs. Experimentally, we could reduce the potential cytotoxicity of DA significantly by stimulating additional leaching of the DA monomers. Other chemicals used to make HCCS were already examined and clarified as nontoxic in our previous study^30^.

After attachment on the coated dishes, rMSCs in the non-coated culture dish showed typical round morphology by representing multiple directional actin filaments and well distributed vinculins. Cells plated on the HCCS-coated dishes demonstrated less spreading with unidirectional actin filaments and limited vinculin distribution. In contrast to the HCCS only group, the majority of rMSCs on the HCCS-PDA-coated dishes displayed morphology similar to that of the control group. This lends evidence to the contribution of DA on enhancing cellular adhesion and attachment toward our biomaterial (Fig. 5C).

DA also had stimulatory effects on proliferation and mineralization of MSCs on HCCS-PDA-coated dishes. Although rMSC growth on HCCS-PDA was significant, it was difficult to distinguish the degree of nodule formation after using osteogenic differentiation media containing full osteogenic factors (10 mM *β*-glycerphosphate, 0.2 mM ascorbic acid, and 10^−4^ mM dexamethasone). As an alternative, we further tested the effect of DA on mineral nodule formation by halving the number of osteogenic factors (*β*-glycerphosphate, ascorbic acid, and dexamethasone). Doing so allowed us to clarify that DA promoted mineralization during 28 days of osteogenic differentiation, as the degree of mineralization was much clearer and enabled us to distinguish between different material-coated dishes. DA treatment without osteogenic factors also formed minor mineral nodules, due to the polymerization of DA monomers. This was also observed from the previous study using MC3T3-E1 cells^23^. In this study, DA receptor blockers were pretreated on MSCs before stimulating with DA to verify the DA receptor-mediated osteogenic effect on rMSCs. The reduced mineral nodule formation showed that DA stimulated osteogenic differentiation on rMSCs through their receptors. After osteogenic differentiation for 14 days, rMSCs showed continued expression of D2 and D4 receptors, though further studies are necessary for more insight to find whether DA can stimulate or inhibit bone mineralization through DA receptor-mediated signal pathways.

After 12 weeks of post-implantation, the entire calvaria was harvested for micro-CT, MAR, and histomorphometric analysis. Both micro-CT and histomorphometric analysis showed that the HCCS-PDA group had higher bone regeneration than the HCCS group without DA content, indicated by greater bone volume and new bone formation (NBF). In micro-CT, the HCCS-PDA group even showed higher bone regeneration as compared to the autograft group (Fig. 7B). Conversely, histomorphometric data revealed that the new bone formation with HCCS-PDA was significantly less than the autograft group (Fig. 8G). This discrepancy is most likely due to the constraints of these analytic techniques concerning biomaterial detection. For example, histomorphometric analysis for NBF percentage determination cannot distinguish between the bone particles and the newly formed bone of the autograft group and thus both are included in the percentage calculation. Yet, the biomaterial particles and newly formed bone are distinguishable with this method, yielding the percentage of NBF for both biomaterial groups to be much lower than that of the autograft group. In other words, only the newly formed bone around the particles in the defect site were quantified for the biomaterial groups while the autograft group quantified the entire explant as well as newly formed bone. Micro-CT detected both new bone and materials as a whole without differentiating newly formed bone from the materials. Thus, the radiopaque result was higher in the biomaterial groups and particularly in the HCCS-PDA group as compared to the autograft group.

The MAR in the defect region of each sample was evaluated, the distribution of the particles led to difficulty in detecting fluorochrome labeling and the data was excluded. Although this data demonstrated higher *in vivo* bone formation with HCCS-PDA particles than HCCS particles in micro-CT and histomorphometric analysis, there is a limitation in evaluating the bone regeneration capability of the whole CSD. It is technically difficult to acquire the entire bone formation in the CSD using the particulate form of grafts with rMSC aggregation, largely due to the available cell delivery methods on the CSD site. Though many studies reported a higher *in vivo* osteogenic effect by using cell aggregates instead of single cells, the material and cell aggregate interaction after implantation remains unclear at present. Further research should also be conducted to obtain more detailed mechanistic insight into the enhanced cellular attachment onto the polydopamine-enhanced surface. Indeed, cell delivery method need to be updated for more vigorous and effective way such as using scaffold with well distributed pores. For future studies, advanced printing technologies can be used as they enable superior precision in controlling the size and internal environment of bone scaffolding, including pore size, connectivity, dimensions, and shape. Fabrication of HCCS-PDA as a patterned, porous scaffold by using 3D printed mold technology can be used to achieve a custom fit design according to the defect size.

## Conclusion

In this study, the novel HCCS-PDA biomaterial was tested for the osteogenic effect on the rMSCs. It was found that HCCS-PDA particulates seeded with rMSC aggregates could enhance cell attachment, proliferation, and mineralization *in vitro* with minimal cytotoxicity. In addition, implantation of these particulates into a rat calvarial critical-sized defect yielded a synergic effect to enhance local bone formation *in vivo*. Here, we confirmed that free DA released from HCCS-PDA had an effect on mineralization by DA receptors expressed on rMSCs. Overall, our results indicate that the HCCS-PDA biomaterial can serve as an osteoconductive scaffolding material, with potential for further development into therapeutic bone tissue engineering strategies and clinical applications.

## Electronic supplementary material

Supplementary data
