# Supplementary material for: Polydopamine-Laced Biomimetic Material Stimulation of Bone Marrow Derived Mesenchymal Stem Cells to Promote Osteogenic Effects
Source: Sci Rep. 2017 Oct 11;7:12984. doi: 10.1038/s41598-017-13326-y (PMC5636820; doi:10.1038/s41598-017-13326-y)
Supplement: Supplementary file 1 — Supplementary data [file 41598_2017_13326_MOESM1_ESM.pdf]

Polydopamine-Laced Biomimetic Material Stimulation of Bone Marrow Derived Mesenchymal Stem Cells to Promote Osteogenesis

Dong Joon Lee[1], Yan-Ting Lee[1], Rui Zou[1], Renie Daniel[2], and Ching-Chang Ko[1,3*]

[1]Oral and Craniofacial Health Sciences Research, School of Dentistry, University of North Carolina, CB #7455, Chapel Hill, NC 27599, USA

[2]Department of Oral and Maxillofacial Surgery, School of Dentistry, University of North Carolina, CB #7454, Chapel Hill, NC 27599, USA

[3]Department of Orthodontics, School of Dentistry, University of North Carolina, CB #7454, Chapel Hill, NC 27599, USA

Supplementary Figure S1

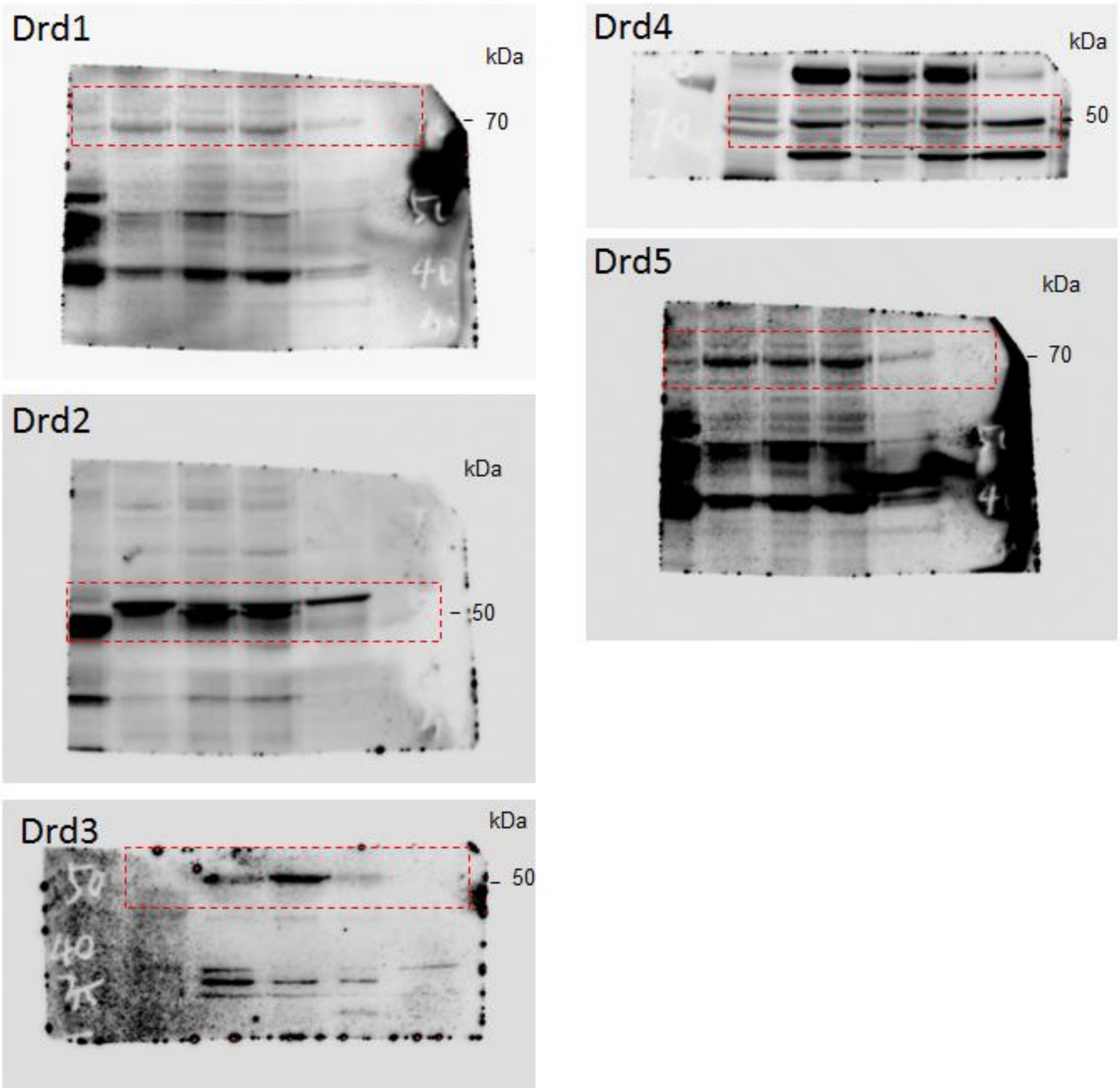

Western Blot analysis of DA receptors expressed on the rMSCs during osteogenic differentiation (From the left lane, mouse brain as control, 0, 4, 7, and 14 days). Each loading was normalized by beta actin (not shown here). The relative molecular weights of each DA receptor were labelled on the right side of the blot image.
